# Regulatory role of *FZP* in the determination of panicle branching and spikelet formation in rice

**DOI:** 10.1038/srep19022

**Published:** 2016-01-08

**Authors:** Xufeng Bai, Yong Huang, Donghai Mao, Mi Wen, Li Zhang, Yongzhong Xing

**Affiliations:** 1 National Key Laboratory of Crop Genetic Improvement and National Center of Plant Gene Research (Wuhan), Huazhong Agricultural University, Wuhan 430070, China; 2 Hubei Collaborative Innovation Center for Grain Industry, Yangtze University, Jingzhou 434025, China

## Abstract

*FRIZZLE PANICLE* (*FZP*) and *RFL*/*ABERRANT PANICLE ORGANIZATION 2* (*APO2*) play important roles in regulating the ABCDE floral organ identity genes. However, the relationships among *FZP* and these floral identity genes in the regulation of panicle formation remain unclear. Here, we used the novel mutant *fzp-11*, wild-type and *FZP*-overexpressing plants to compare the expression of these genes during panicle development by real-time PCR and *in situ* hybridization. The results indicate that *FZP* is a major negative regulator of *RFL*/*APO2* and determines the transition from panicle branching to spikelet formation. Moreover, overexpression of *FZP* severely represses axillary meristem formation in both the vegetative and reproductive phases and the outgrowth of secondary branches in panicle. *FZP* overexpression positively regulates the expression of a subset of the class B genes, *AGL6* genes (*OsMADS6* and *OsMADS17*) as well as class E genes (*OsMADS1*, *OsMADS7* and *OsMADS8*) in floral meristem (FM). Thus, it suggested that *FZP* could specify floral organ identity by regulating the related OsMADS-box genes.

Rice is an important model that is used to study plant growth and development. Panicle formation, including panicle branching and spikelet formation, is an integral process in rice development that determines grain yield. The emergence and growth of the lateral organs, such as the leaf, tiller and panicle branch, is accomplished by axillary meristem initiation and elongation, which are important events in the formation of plant architecture. After the transition from vegetative to reproductive growth, the rice panicle meristem forms and differentiates into the panicle axis, panicle branches and spikelets. Panicle branching involves either primary branching from the panicle axis or secondary branching from the primary branches. Both primary and secondary branches bear spikelets, the number of which is an important determinant of grain yield.

*FRIZZY PANICLE* (*FZP*) can repress panicle branching and/or positively influence floral meristem identity^1^. The FZP protein, which contains an APETALA2/ETHYLENE RESPONSE FACTOR (AP2/ERF) domain, is an ortholog of the maize transcription factor BRANCHED SILKLESS1 (BD1)^2^. A total of 11 *fzp* mutant alleles (*fzp-1* to *fzp-10* and *BRANCHED FLORETLESS 1* (*bfl1*)) were identified through efforts involving ethyl methanesulfonate (EMS) mutagenesis, γ-ray mutagenesis, screening of *Ac* transgenic pools, *Ds* tagging and spontaneous mutation^1^,^2^,^3^,^4^. All the mutants produce more secondary and high-order branches rather than normal spikelets^1^,^3^. In rice, several genes positively regulate panicle branching. RNAi-induced silencing of *RFL*/*ABERRANT PANICLE ORGANIZATION 2* (*APO2*), the rice homolog of *FLORICAULA* (*FLO*) from *Antirrhinum* and *LEAFY* (*LFY*) from *Arabidopsis thaliana*^5^, severely reduced the number of the primary branches^6^. *LAX PANICLE1* (*LAX1*) encodes a basic helix-loop-helix (bHLH) transcription factor, which is required for the initiation and maintenance of axillary meristems in rice panicles. There are fewer primary branches in the panicle of *lax1* mutants^7^. *SMALL PANICLE* (*SPA*), which promotes panicle branching^8^, was further proved to be an allele of *MONOCULM 1* (*MOC1*)^9^. The panicles of *lax1 spa* double mutants are wire-like structures with no branches^8^. In addition to these genes, two quantitative trait loci (QTLs), *DENSE and ERECT PANICLE 1* (*DEP1*) and *GRAIN NUMBER 1a* (*GN1a*), also control panicle branching. *Gn1a* encodes a cytokinin oxidase/dehydrogenase that regulates the number of secondary branches by affecting the accumulation of cytokinin in the panicle meristem^10^. *DEP1* encodes a protein that shares some homology with the N-terminus of the protein encoded by *GS3* (a major QTL for grain shape in rice), which simultaneously controls the number of the primary and secondary branches^11^. Nakagawa *et al.* reported that overexpression of *RCN1* and *RCN2* (putative *TERMINAL FLOWER1* (*TFL1*)/*CENTRORADIALIS* (*CEN*) orthologs in rice) delays the transition from vegetative to reproductive growth, increases the number of secondary branches and may cause formation of tertiary branches^12^.

It is reported that the ABCDE model governs the flower development in *Arabidopsis*^13^,^14^,^15^. Flower development is mainly regulated by MADS-box genes, with the exception of the class A gene *APETALA2* (*AP2*)^13^,^15^,^16^,^17^. In rice, the OsMADS-box genes also play key roles in regulating floral morphology^18^,^19^,^20^,^21^,^22^,^23^,^24^,^25^,^26^. Moreover, *RFL*/*APO2* was reported to negatively regulate floral meristem (FM) formation together with *APO1*^27^. Although *FZP*, *RFL*/*APO2* and OsMADS-box genes play important roles in FM determination and normal spikelet formation, the regulatory relationships involved remain poorly understood. In this study, we compared the expression of genes controlling panicle architecture and floral organs in plants of overexpressing *FZP*, wild-type (Dongjin, DJ) and the new mutant *fzp-11*, and we concluded that *FZP* regulates panicle branching and spikelet formation by regulating *RFL*/*APO2* and *FZP* overexpression elevated the expression of some floral identify genes.

## Results

### Identification of the mutant *fzp-11* and complementation tests

**Figure 1:**
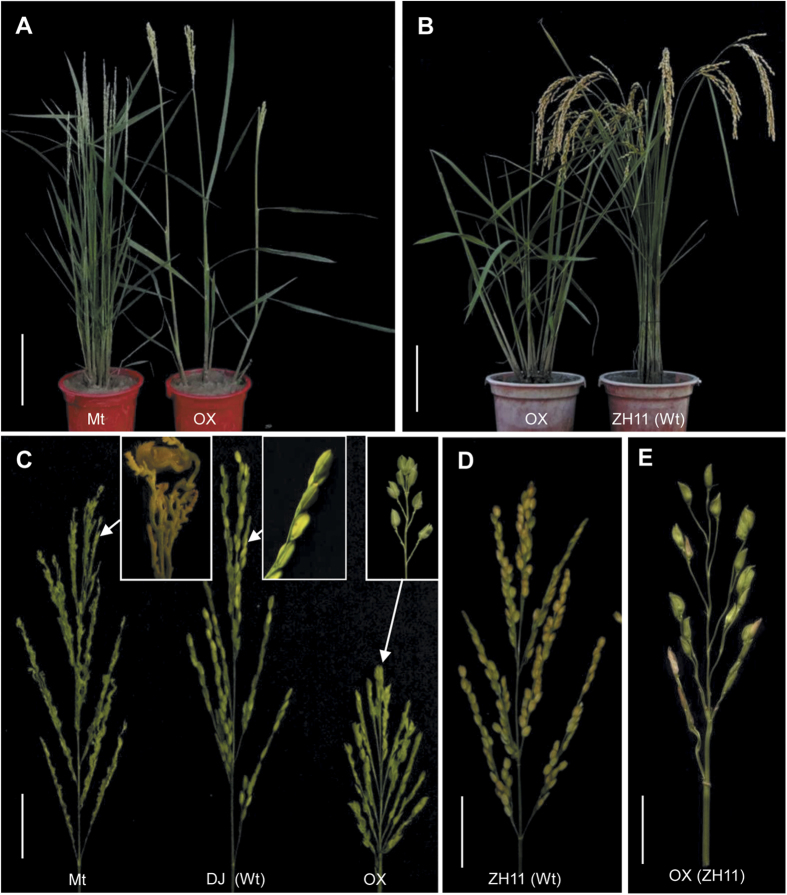
Plant status and panicle architecture of the mutant *fzp-11*, wild-type and *FZP*-overexpressing plants. (**A**) Mature mutant (Mt) *fzp-11* (left) and OX*-FZP-*(*fzp-11*) (right) plants. (**B**) A wild-type (ZH11) plant (right) and OX*-FZP-*(ZH11) plant (left). The photo was taken when the control Zhonghua 11 reached maturity. (**C**) Panicles of the mutant *fzp-11* (left), wild-type (Dongjin (DJ), middle) and OX-*FZP-*(*fzp-11*) (right) plants. (Insets) Magnified view of the primary branches of *fzp-11*, DJ and OX-*FZP-*(*fzp-11*). (**D**) Mature panicle of the control Zhonghua 11. (**E**) Panicle of OX*-FZP-*(ZH11). Scale bars = 15 cm (A and B), 5 cm (**C** and **D**), 3 cm (**E**).

The phenotype of the *fzp-11* mutant was similar to those of the previously reported *fzp* mutants. The panicle morphology of *fzp-11* was significantly different from that of wild-type DJ, with numerous panicle branches, including higher order tertiary and quaternary branches that replaced the normal spikelets in primary and secondary branches, whereas they were normally not produced in wild-type DJ plants (Fig. 1C and Table 1). Sporadically bare terminal spikelets with abnormal floral organs were produced from the panicles of *fzp-11* plants. Thus, *fzp-11* was regarded as a mutant of *FZP*. Comparative sequencing of the wild-type allele *FZP* and mutant allele *fzp-11* showed that a single nucleotide polymorphism (SNP) involving an A to T mutation in the region encoding the AP2/ERF domain caused an Asp to Val amino acid substitution in *fzp-11* plants (Figure S1A,B). The SNP alleles of 142 plants co-segregated with their panicle phenotypes: all DJ (A) and heterozygous (A/T) plants had normal spikelets and all *fzp-11* (T) plants produced abnormal panicles. Progeny tests showed a segregation ratio of 34:75:33 for homozygous DJ (A), heterozygous (A/T) and homozygous *fzp-11* (T) plants, which is consistent with the expected segregation ratio of a single Mendelian factor (χ^2^ = 0.46, *P *> 0.05). Further, the complementary plasmid containing the functional *FZP* was introduced into *fzp-11* homozygous plants. Six independent positive transgenic plants (T_0_) were obtained, which showed complete complementation of the *fzp-11* phenotype (Figure S1C). These data support the finding that *fzp-11* is a novel *FZP* mutant.

### Overexpression of *FZP* in *fzp-11* and ZH11

**Figure 2:**
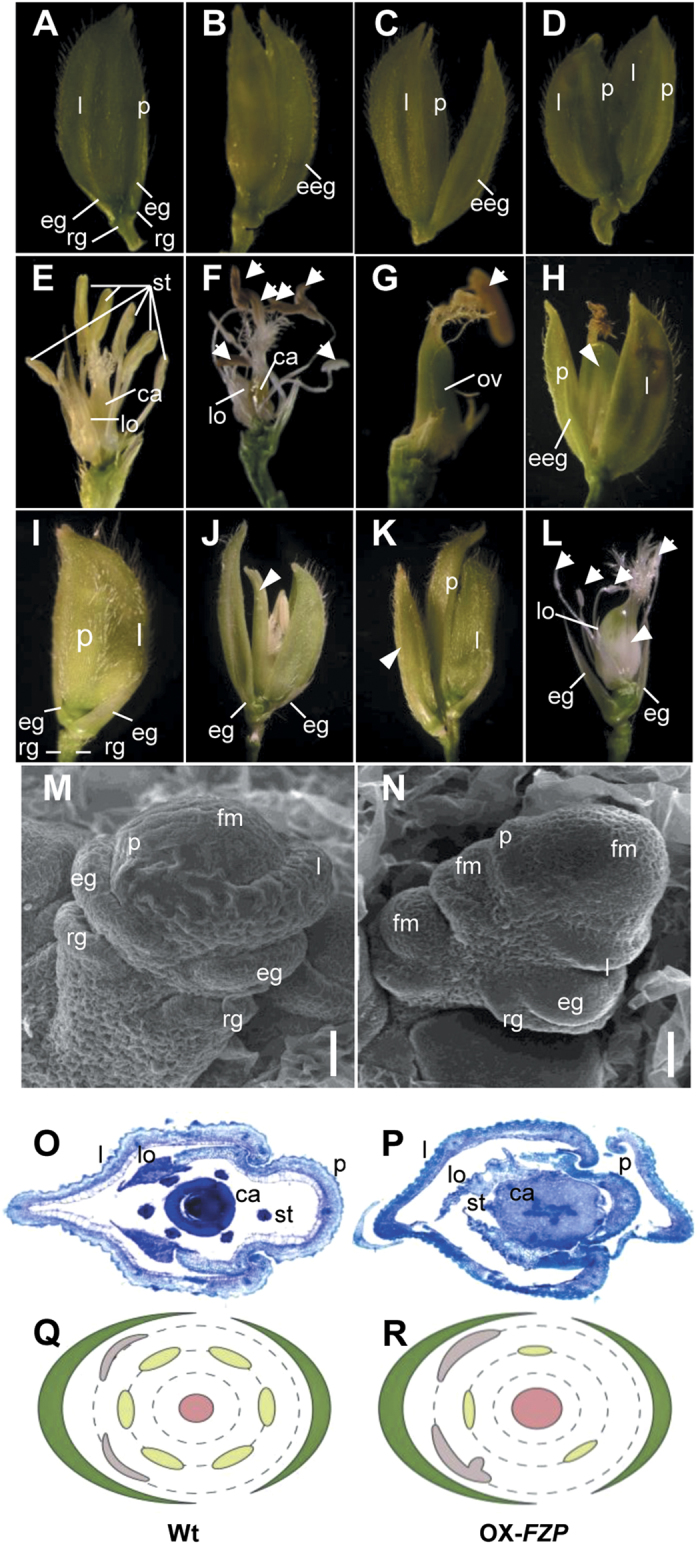
Phenotypes of spikelets and floral organs in the OX-*FZP*-(*fzp-11*) (A–H,M–R) and OX-*FZP*-(ZH11) plants (I–L). (**A**) Spikelet with a normal lemma and palea but sterile stamen. (**B**) Abnormal spikelet with an elongated empty glume (eeg) similar to a lemma. (**C)** Abnormal spikelet with an eeg similar to a palea. (**D**) Additional ectopic spikelet formed at the end of the branch. (**E**–**H**) Different stages of spikelet and/or floral organs after heading. The white arrows indicate abortive stamens; the white arrowhead indicates an enlarged ovary/carpel. (**I**) Spikelet with a normal lemma and palea but sterile stamen. (**J**,**K**) Abnormal spikelets. White arrowheads indicate enlarged lodicules (**J**) and an ectopic spikelet (**K**). (**L**) Floral organs. White arrows indicate abnormal stamens, and the white arrowhead indicates an enlarged ovary/carpel. (**M**,**N**) Scanning electron microscopy images of wild-type (DJ) and OX-*FZP*-(*fzp-11*) spikelets in the early developmental stage, Scale bars = 20 μm. (**O**,**P**) Transverse sections of flowers from wild-type (DJ) and OX-*FZP*-(*fzp-11*). (**Q** and **R**) Diagrams of wild-type and OX-*FZP* flowers. The lemma and palea are indicated in dark green; lodicules, stamens and carpels are indicated in gray, light green and pink, respectively. Abnormal lodicules, fewer stamens and enlarged carpels were observed in the flowers of OX-*FZP* plants. The lemma and palea were artificially removed in (**E**–**G**),(**L**). l, lemma; p, palea; eg, empty glume; rg, rudimentary glume; egg, elongated empty glume; st, stamen; ca, carpel; lo, lodicule; ov, ovary; fm, floral meristem.

The OX*-FZP*-(*fzp-11*) (T_0_) transgenic plants, which were obtained by transforming *fzp-11* plants with the construct *p35S*::*FZP*_Nip_, had fewer tillers, shorter and more abnormal panicles with fewer secondary branches (without tertiary branches) and only terminal spikelets in most primary branches (Fig. 1A,C and Table 1). It is surprising that panicle branching was so dramatically reduced in the transgenic plants (Table 1). However, there were a few node-like vestiges in the primary branches where secondary branches are normally produced in wild-type plants, indicating that constitutive overexpression of *FZP* severely represses the outgrowth of secondary branches. In addition, OX-*FZP*-(*fzp-11*) plants had a series of defects in spikelet structure (Fig. 2A–D). Lemma-like and palea-like organs were produced due to the elongated empty glumes (Fig. 2B,C). Most noticeably, double terminal spikelets were also observed at the ends of some primary branches, whereas one terminal spikelet formed in the wild-type plants (Fig. 2D). The ectopic formation of FM was observed in the OX-*FZP*-(*fzp-11*) plants (Fig. 2M,N), which might result in the additional terminal spikelet in primary branches. The OX-*FZP*-(*fzp-11*) plants failed to yield seed owing to defects in the number and status of stamen; frequently, enlarged lodicules and ovaries/carpels were observed in the spikelets of these plants (Fig. 2E–H,O–R and Table 2). In addition, the same construct (*p35S*::*FZP*_Nip_) was transformed into ZH11. OX-*FZP*-(ZH11) (T_0_) transgenic plants showed delayed heading and fewer tillers. Similar to OX-*FZP*-(*fzp-11*) plants, OX-*FZP*-(ZH11) plants had fewer secondary panicle branches than the wild-type (ZH11) plants, and some had none (Fig. 1D,E). Meanwhile, OX-*FZP*-(ZH11) plants also had defects in floral organs, including double terminal spikelets, fewer stamen, sterile stamen, elongated palea, enlarged lodicules and enlarged ovaries/carpels (Fig. 2I–L and Table 2). Thus, filled grain could also not be harvested from the OX-*FZP*-(ZH11) plant.

Both OX-*FZP*-(*fzp-11*) and OX-*FZP*-(ZH11) plants had large tiller and leaf angles, dark green leaf blades and thick stems (Fig. 1A,B and Figure S2A–F). In transverse stem sections, there were more layers of larger cells in the transgenic positive plants than in the wild-type (ZH11) plants, accounting for the increase in stem thickness (Figure S2C,D). Differences in the adaxial surface were observed in pulvinar cross-sections between the wild-type (ZH11) and positive plants. The adaxial surface of the positive plants was more plane than that of wild-type plants (Figure S2F). OX-*FZP*-(*fzp-11*) plants had fewer roots than *fzp-11* plants (Figure S2G and Table 1). Thus, overexpression of *FZP* in cultivated rice represses axillary meristem formation in both the vegetative and reproductive phases and results in a reduction in the numbers of roots, tillers and panicle branches. Besides, the apical meristem of the inflorescence in OX-*FZP*-(*fzp-11*) plants was pre-degenerated, the primary branch meristem (PBM) was more flat compared with those of wild-type (DJ) plants (Figure S2H,I).

### *FZP* represses the expression of *RFL*/*APO2*

**Figure 3:**
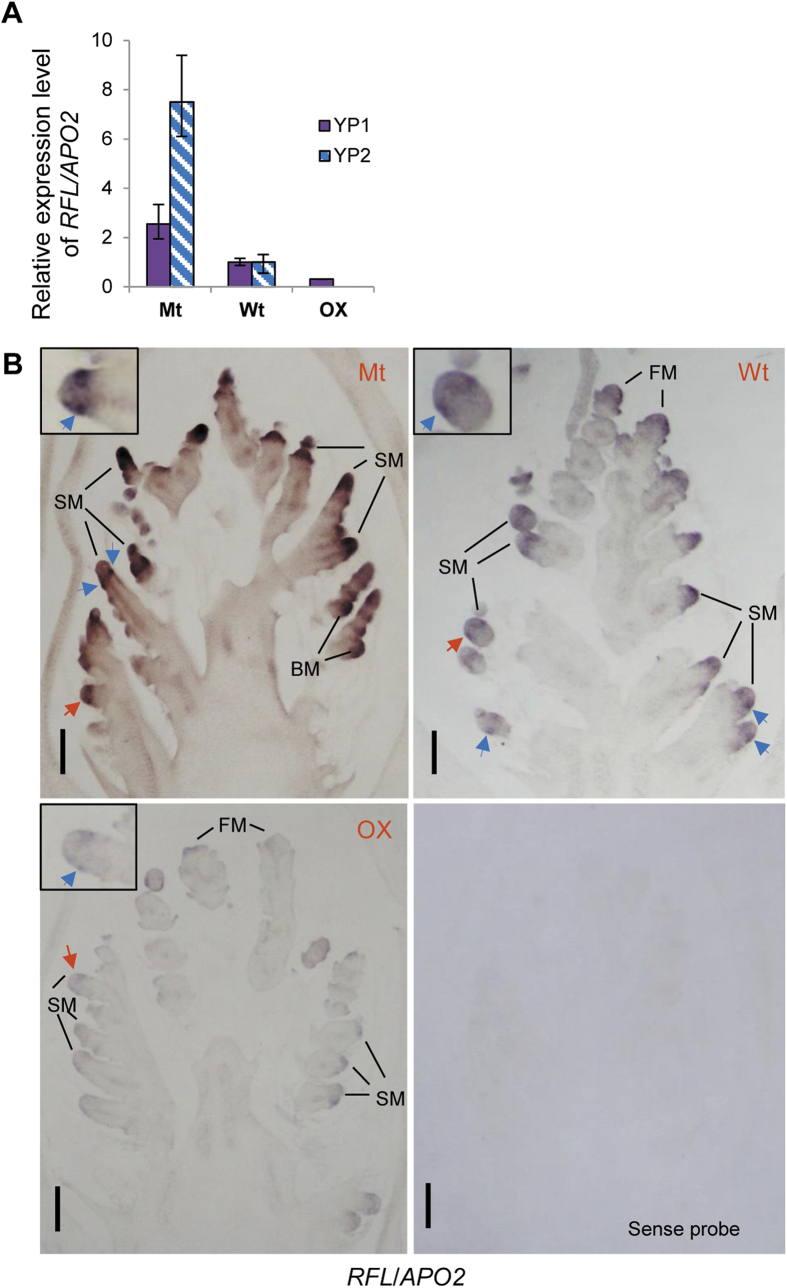
Expression patterns of *RFL/APO2*. (**A**) The relative expression levels of *RFL*/*APO2* in the YP1and YP2 of plants from *fzp-11*, wild-type (DJ) and OX-*FZP*-(*fzp-11*) plants. *Ubiquitin* served as the control. The bars indicate standard deviations. (**B**) RNA *in situ* hybridization analysis of *RFL*/*APO2*. The YP1 of Mt (*fzp-11*), Wt (DJ) and OX (OX-*FZP*-(*fzp-11*)) showing branch meristems (BM), spikelet meristems (SM) and floral meristem (FM) were used for expression analysis. The SM marked with red arrows was closed-up in the insets, blue arrows indicate areas of expression, sense probe control is also showed. Scale bar = 100 μm.

**Figure 4:**
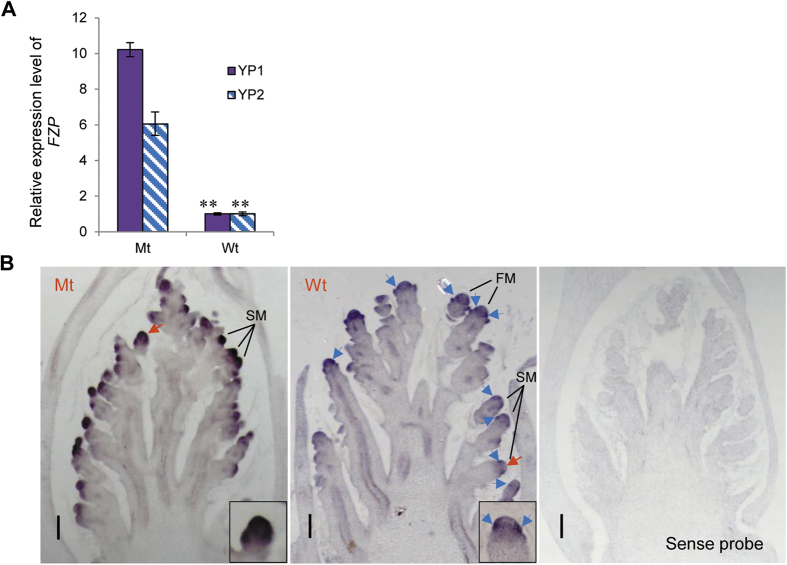
Expression patterns of *FZP.* (**A**) Relative expression levels of *FZP* in the YP1 and YP2 of mutant *fzp-11* and wild-type (DJ) plants. *Ubiquitin* served as the control. The bars indicate standard deviations. **P < 0.01 (Student’s *t*-test). (**B**) RNA *in situ* hybridization analysis of *FZP*. The YP1 of Mt (*fzp-11*) and Wt (DJ) showing spikelet meristems (SM) and floral meristem (FM) were used for expression analysis. The SM marked with red arrows was closed-up in the insets, blue arrows indicate areas of expression, sense probe control is also showed. Scale bar = 100 μm.

Expression of the genes involved in panicle branching *(GN1a*, *DEP1*, *RCN1*, *LAX1*, *RFL*/*APO2*) was monitored respectively in the 1.5–2.5-mm-long panicles (YP1) and 1-cm-long panicles (YP2) of the *fzp-11*, wild-type DJ, and OX-*FZP*-(*fzp-11*) plants by real-time PCR. We found the transcription of *RFL*/*APO2* in YP1 and YP2 showed the highest expression in *fzp-11* plants, the lowest expression in the OX-*FZP*-(*fzp-11*) plants, and medium in the wild-type DJ plants (Fig. 3A). The expression of *LAX1* in YP2 was higher in *fzp-11* than that in wild-type DJ, whereas its expression in YP1 showed no difference among *fzp-11*, wild-type DJ, and OX-*FZP*-(*fzp-11*) (Figure S3A). To further compare expression patterns of *RFL*/*APO2* and *LAX1* among three genotypes (*fzp-11*, wild-type DJ and OX-*FZP*-(*fzp-11*)), we performed *in situ* hybridization of *RFL*/*APO2* in BM (branch meristem), SM (spikelet meristem) and FM (floral meristem) and of *LAX1* in BM where *LAX1* mainly expressed. They showed the results similar to those of qRT-PCR (Fig. 3B and Figure S3B). Besides, microarray analysis showed that *RFL*/*APO2* in freshly headed panicles (FHP) of *fzp-11* plants was upregulated 9-fold relative to DJ plants (Table S2). These results indicated that *FZP* significantly represses the expression of *RFL*/*APO2*. To further examine the expression suppression of *FZP* to *RFL*/*APO2*, we compared the expression levels of *RFL*/*APO2* in YP1 of OX-*FZP*-(ZH11) and control plants using real-time PCR. As expected, the expression level of *RFL*/*APO2* was much lower in the OX-*FZP*-(ZH11) plants (Figure S4A). This result is consistent with the reduced branching observed in the OX-*FZP*-(ZH11) plant (Fig. 1D,E). Thus, the mechanism by which *FZP* represses panicle branching may involve a decrease in the expression of *RFL*/*APO2*. Interestingly, the expression level of *FZP* was dramatically increased in young panicles of *fzp-11* plants compared with young panicles of DJ plants based on real-time PCR and *in situ* hybridization analysis (Fig. 4A,B).

### Expression analysis of genes involved in the ABCDE model

Several OsMADS-box family genes specify different floral organ identities according to the ABCDE model of flower development. Mutation of any of these genes causes substantial defects in floral organs. Here, the expression of the related OsMADS-box genes was examined by qRT-PCR in YP1, YP2 and FHP collected from DJ and *fzp-11*, respectively. The results revealed that the class B genes (*OsMADS2*, *OsMADS4* and *OsMADS16*), the class C genes (*OsMADS3* and *OsMADS58*), the class E genes (*OsMADS1*, *OsMADS7* and *OsMADS8*) and two *AGL6* genes (*OsMADS6* and *OsMADS17*) were downregulated in the *fzp-11* plants relative to wild-type DJ plants (Figure S4B). It was corresponding to the results of microarray analysis (Table S2). Notably, the expression of *OsMADS7* was 120-fold lower in *fzp-11* plants than in wild-type DJ plants. Additionally, *SNB* (*SUPERNUMERARY BRACT*), an *AP2* family gene regulating the transition from SM to FM^28^, was slightly downregulated in YP1 and YP2 of *fzp-11* plants (Figure S4B).

**Figure 5:**
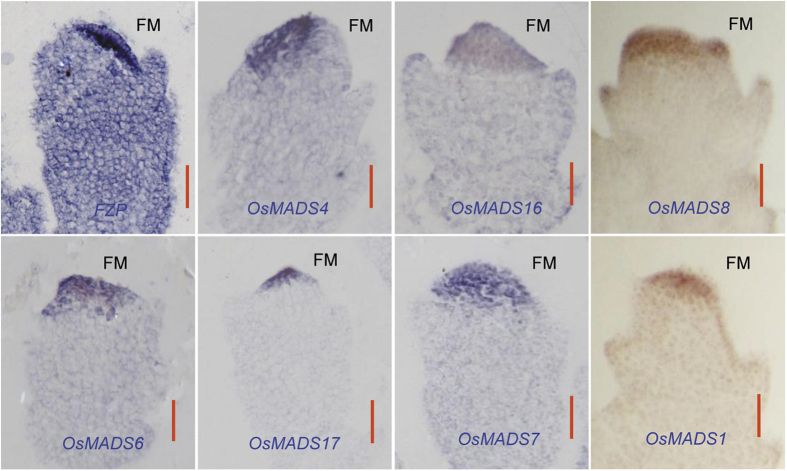
RNA *in situ* hybridization analysis of *FZP* and OsMADS-box genes. The floral meristems (FM) from YP1 of OX (OX-*FZP*-(*fzp-11*)) plants were used for expression analysis of *FZP* and the related flower identity genes. 7 OsMADS-box genes were identified ectopic and excessive expression in the FM. Scale bar = 20 μm.

Defects in some floral organs were observed in both OX-*FZP*-(*fzp-11*) and OX-*FZP*-(ZH11) plants. The fewer and weaker stamens and abnormal carpels/ovaries resulted in their spikelet sterility. Thus, the expression of the related floral identity genes was investigated by *in situ* hybridization in the FM of YP1 in OX-*FZP*-(*fzp-11*) plants. The results indicated that seven of them, *OsMADS4* and *OsMADS16* (class B genes), *OsMADS6* and *OsMADS17* (*AGL6* genes), *OsMADS1*, *OsMADS7* and *OsMADS8* (class E genes) were ectopically and abundantly expressed in the FM compared with the expression of these floral identity genes in wild-type plants (Figure S5) and previous reports^29^, where *FZP* is also highly expressed (Fig. 5). In contrast, the expression of class C genes (*OsMADS3* and *OsMADS58*) was barely detectable.

### Co-expression analysis of *FZP*, *RFL*/*APO2* and the floral identity genes

To confirm whether an overlapping expression pattern exists between *FZP* and the other genes investigated in this study, we analyzed the expression patterns of *FZP*, *RFL/APO2* and the floral identity genes in the *japonica* variety Nipponbare from the database (http://ricexpro.dna.affrc.go.jp/Zapping)^30^,^31^. The expression pattern of *FZP* was very similar to that of *FRL*/*APO2*, which was co-expressed in the younger panicles (from 0.6–4 mm). The expression of *RFL*/*APO2*, *FZP* and the flower identity genes was over-lapped in 3–4-mm-long young panicles (http://ricexpro.dna.affrc.go.jp/Zapping). Additionally, RNA *in situ* hybridization detected the expression of the partial floral identity genes in the FM emergence stage (YP1) in DJ. The results showed that the genes within the same class shared a very similar expression pattern. For example, the class B genes expressed in the similar region in FM (Figure S5). Surprisingly, besides in SM, *FZP* also strongly expressed in specific region of FM, where class B genes expressed (Fig. 4B and Figure S5). To confirm this finding, we performed *in situ* hybridization of *FZP* with more than three biological repeats and got the consistent result that *FZP* expressed both in SM and FM (Fig. 4B and Figure S3C). Besides, *RFL*/*APO2* has a similar expression pattern to *FZP* in SM (Figs 3B and 4B). However, *RFL*/*APO2* has a wider expression region in the FM that partly overlapped with the expression regions of the other investigated genes (Figure S5). On the other hand, a co-expression pattern was also observed among *OsMADS4*, *OsMADS16*, two *AGL*6 genes, *OsMADS1*, *OsMADS7* and *OsMADS8*, and *FZP* in the FM of YP1 in OX-*FZP*-(*fzp-11*) plants (Fig. 5). Thus, the co-expression of these genes provides them a chance to interact at the transcriptional level.

## Discussion

In *Arabidopsis*, class A genes function in controlling the sepal of whorl 1 and restrict the activation of class C genes via the actions of two genes: *APETALA1* (*AP1*) and *AP2*/*ERF*, of which *AP1* is a MADS-box gene encoding transcription factors^32^,^33^. However, the function of class A genes in floral organ specification is still debated due to reports that class A genes are absent in other species^26^. Recently, an (A)BC model was proposed that defined class (A) function as follows: (1) Class (A) genes are expressed before class B and C genes and act to establish FM identity, and (2) class (A) genes are required for the later activation and regulation of class B and C genes^14^,^34^. *FZP* encodes an AP2/ERF transcription factor and enables the establishment of FM identity^1^. In this study, we found that the downregulation of class B and C genes accompanied with abortion of spikelet formation in *fzp-11* plants; the expression of *FZP* was initiated before the expression of class B and C genes. *In situ* hybridization indicated that *FZP* expressed in the FM of wild-type, and co-expressed with the class B genes (Figure S5). In parallel, *FZP* participates in expression regulation of class B and other floral identity genes in *FZP*-overexpressing plants, excessive and ectopic expression of *FZP* in the FM promoted class B genes (*OsMADS4* and *OsMADS16*), *AGL6* (*OsMADS6* and *OsMADS17*) and class E genes (*OsMADS1*, *OsMADS7* and *OsMADS8*). The change of their expression could result in a series of defects in flower structure. For example, the additional lemma-like, palea-like organs and additional terminal spikelets produced in *FZP*-overexpressing plants (Fig. 2B–D,K). Furthermore, the ultrastructure of the early developmental spikelet in OX-*FZP*-(*fzp-11*) showed that the ectopic FM was formed nearby the terminal FM, which probably results in formation of additional terminal spikelet (Fig. 2M,N).

*RFL*/*APO2* was previously implicated in the regulation of panicle branching^5^. A reduction in panicle branching was observed in both RNAi-*RFL*/*APO2* and *apo2* plants^6^,^27^. Additionally, the *lax1* mutants had significantly reduced primary branches^7^. Thus, both *RFL*/*APO2* and *LAX1* positively regulate panicle branching. In contrast, *FZP* was reported to repress panicle branching^1^. It is unclear how these three genes coordinate to regulate panicle branching. The expression patterns of the three genes have been individually reported in separate studies^1^,^5^,^7^,^8^,^27^,^35^. However, their relationship at the transcriptional level is less well defined. Rao *et al.* reported that *LAX1* is downregulated and *FZP* is upregulated in young panicles of RNAi-*RFL*/*APO2* plants^6^, it seems that both *RFL*/*APO2* and *LAX1* antagonize *FZP* at the transcriptional level. However, it is not clear whether *RFL*/*APO2* or *LAX1* is the player that acts to antagonize *FZP*. In this study, *FZP* showed a clear co-expression with *RFL*/*APO2* in SM, and antagonized to *RFL*/*APO2* at the transcriptional level (Figs 3A,B and 4B and Figure S3B). However, there was no significant difference of *LAX1* expression level in YP1 by qRT-PCR and in BM by *in situ* hybridization among *fzp-11*, wild-type (DJ) and OX-*FZP*-(*fzp-11*) plants (Figure S3A,B). These results suggest that *RFL*/*APO2* antagonizes *FZP* independently of *LAX1*, and the coordinated expression of them in the SM of wild-type plants activates the transition from the SM to the FM. Once the transition is complete, the panicle branching is ceased. Thus, overexpression of *FZP* in the SM accelerates the transition from the SM to the FM, resulting in a short panicle with fewer branches. On the contrary, the *fzp* loss-of -function mutant produces panicles with numerous branches.

**Figure 6:**
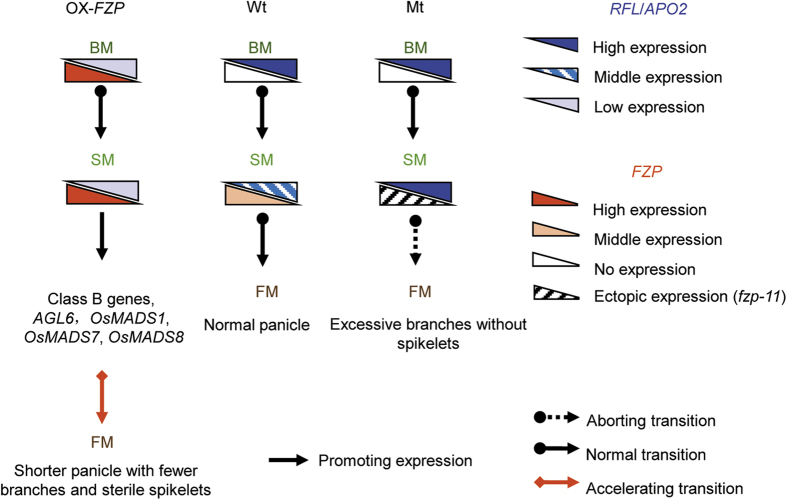
The proposed regulatory model of *FZP* and *RFL*/*APO2* for rice panicle branching and the floral organ identity determination.

*PUCHI*, an ortholog of *FZP* in *Arabidopsis*, is required for FM identity and has been reported to promote expression of *LFY*^36^. Moreover, *LFY* and *SEP3* function together to activate the expression of class B and C genes in *Arabidopsis*^37^. In rice, *RFL*/*APO2* is required for class C gene activity^27^, while *FZP* is required for the transition from SM to FM^1^. However, in this study, *FZP* repressed *RFL*/*APO2*, whereas *PUCHI* was previously found to promote the expression of *LFY* in *Arabidopsis. PUCHI* may positively regulate class B and C genes via *LFY*^36^,^37^, whereas excessive and ectopic expression of *FZP* in FM could promote the expression of class B genes independent of *RFL*/*APO2*. However, the class C genes was not be detected that may be attributed to the repression of *RFL*/*APO2* in OX-*FZP*-(*fzp-11*) plants. We have proposed a model to illustrate the network that regulates FM determination in rice by integrating data obtained from three genotypes (Mt (*fzp-11*), Wt (Dongjin) and OX-*FZP*) (Fig. 6). Proper temporal and spatial expression of both *FZP* and *RFL*/*APO2* is required for FM identity, and each gene fine-tunes the expression level of the other gene to regulate the transition from SM to FM in wild-type plants that produce panicles with normal architecture. In the mutant (e.g., *fzp-11*), the transition from SM to FM was abolished, which resulted in branches without spikelets. Excessive expression of *FZP* in the FM, where *RFL*/*APO2* is severely repressed, enhances the expression of class B genes, *AGL6* genes and class E genes to accelerate the formation of FM, and accompanied with the absent expression of class C genes. The disordered expression pattern and changed expression level of these flower identity genes results in the phenotype of shorter panicles with fewer branches and sterile spikelets. This also suggested *FZP* could participate in the regulation of the expression of the class B, C, *AGL6* and class E genes. However, whether the direct regulation between *FZP* and these OsMADS-box genes was still unclear, which need to be further validated in the future.

## Materials and Methods

### Plant Materials

A spontaneous mutant, *fzp-11*, was identified in the T-DNA insertion mutant line PFG_1B-11535. The genetic background of this T-DNA line is Dongjin (DJ) (*Oryza sativa* ssp. *japonica*) (http://signal.salk.edu/cgi-bin/RiceGE)^38^. A heterozygous (*FZP/fzp-11*) plant was used to produce an F_2_ population, comprising 142 plants, for analysis of co-segregation. A total of 109 F_3_ families (wild-type DJ and heterozygous plants) were planted to analyze the progeny because plants with the genotype *fzp-11/fzp-11* could not produce seeds.

The *FZP* gene was overexpressed in *fzp-11* and a cultivar Zhonghua11 (ZH11) plants. Both young panicles and freshly headed panicles (FHP) from *fzp-11*, wild-type and *FZP*-overexpressing plants (T_0_) were used for expression analysis.

### Sequencing analysis

Fresh leaves were collected from each genotype, and the CTAB method was used to extract genomic DNA^39^. The entire ORF of *FZP* was amplified from genomic DNA by PCR using Ex-Taq (Takara, Otsu, Japan). All PCR analysis was conducted using standard PCR protocols with GCI buffer (Takara, Otsu, Japan). For sequencing, 5 μl of PCR product was digested by simultaneous incubation with ExoI (5 units) and shrimp alkaline phosphatase (0.26 units) in 1 × PCR buffer at 37 °C for 1 h followed by 80 °C for 20 min. Each PCR fragment was sequenced three times. Sequences were assembled using SEQUENCHER 4.1.2 (Gene Codes Corporation, Ann Arbor, MI, USA).

### Vector construction and transformation

Sequencing analysis showed that there is no polymorphism across *FZP* among DJ, Zhonghua11 and Nipponbare, and Nipponbare BAC (Bacterial Artificial Chromosome) library was available in our lab. Therefore, an 8.3-kb genomic DNA fragment containing the entire *FZP* coding region, the 6.5-kb 5′upstream sequence, and the 800-bp 3′ downstream sequence was isolated from Nipponbare BAC (A0044I19) by restriction enzymes SpeI and PstI. Then it was cloned into the binary vector pCAMBIA1301 for complementation tests. The open reading frame (ORF) of *FZP* from Nipponbare was amplified by PCR using the primers COZP. The genomic fragment was cloned into the vector pCAMBIA1301S such that expression of the ORF from Nipponbare was driven by the cauliflower mosaic virus 35S promoter (*p35S*::*FZP*_Nip_).

Callus was induced from seeds harvested from the *FZP*/*fzp-11* plants. The genotypes of calli from hundreds of seeds were individually examined by PCR and sequenced using S3 primers (Figure S1B; Table S1). Calli with an *fzp-11/fzp-11* genotype (mutant, DJ genetic background) were collected for subsequent transformation with the complementary vector and the construct *p35S*::*FZP*_Nip_. In addition, callus from *japonica* rice ZH11 was transformed with the construct *p35S*::*FZP*_Nip_. The *p35S*::*FZP*_Nip_ construct was introduced into *Agrobacterium tumefaciens* strain EHA105, which was used to transform calli as previously described^40^.

### Expression analysis

#### qRT-PCR and microarray assay

Total RNA was extracted from both YP1 and YP2 (1.5–2.5-mm-long and 1-cm-long panicles), and FHP using Trizol reagent (Invitrogen, California, USA) and subsequently used for real-time PCR and microarray analyses. Quantitative analysis of gene expression was performed using SYBR Premix Ex Taq (TaKaRa, Otsu, Japan) and an Applied Biosystems 7500 Real-Time PCR System. The data were analyzed using the relative quantification method^41^, and the rice *ubiquitin* gene (LOC_Os03g13170) was used as an internal control. All assays were performed with three biological and technological repeats. The Capitalbio Corporation (www.capitalbio.com, Beijing, China) performed the microarray experiments and data analysis. Relevant PCR primer sequences are provided in Table S1.

#### RNA *in situ* hybridization

Specific fragments of the 13 examined genes were amplified with the primer pairs listed in Table S1. The products were then inserted into the pGEM-T vector (Promega, Madison, USA) for RNA transcription *in vitro*. The respective sense and antisense probes were produced using SP6 and T7 transcriptase labeled with digoxigenin (Roche, Mannheim, Germany). Plant tissues were collected and fixed in FAA solution (50% ethanol, 5% acetic acid and 3.7% formaldehyde) at 4 °C overnight after vacuum. RNA *in situ* hybridization and immunological detection were carried out as described previously^42^.

### Histological analysis and scanning electron microscopy observation

Tissues were collected and fixed in FAA (50%) for over-night and dehydrated in a series of graded ethanol. The tissues were further substituted by xylene and embedded in paraplast plus (Huayong, Shanghai, China). They were cut into 7-μm thick sections, stained with toluidine blue and observed using a light microscope. Transverse sections were photographed using a Nikon Eclipse 80i microscope. SEM observation was observed with a scanning electron microscope (JSM-6390LV, JEOL, Akishima-shi, Japan), as described previously^43^.

## Additional Information

**How to cite this article**: Bai, X. *et al.* Regulatory role of *FZP* in the determination of panicle branching and spikelet formation in rice. *Sci. Rep.* **6**, 19022; doi: 10.1038/srep19022 (2016).

## Supplementary Material

Supplementary Information

## Figures and Tables

**Table 1:** The related phenotypic values in the plants of *fzp-11*, Wt (DJ) and OX-*FZP-*(*fzp-11*).

Traits	*fzp-11*(M ± SD)	Wt (DJ)(M ± SD)	OX-*FZP*-(*fzp-11*)(M ± SD)
No. of roots	51 ± 15	48 ± 13	34 ± 11**
Panicle length (cm)	18.0 ± 3.0	18.3 ± 2.7	8.5 ± 1.1**
Flag leaf length (cm)	39.0 ± 14.8	37.2 ± 16.0	21.3 ± 4.7**
Flag leaf width (cm)	1.31 ± 0.10	1.29 ± 0.15	1.48 ± 0.16**
No. of primary branch	9.9 ± 2.2	10.3 ± 2.0	9.8 ± 2.1
No. of secondary branch	38 ± 9.1	23.3 ± 5.6	5.2 ± 2.7**
Spikelets per panicle	0	100 ± 6	53 ± 19**

M ± SD, mean ± standard deviation. ** Significantly different between the plants of OX-*FZP* (*fzp-11*) and wild-type (DJ), and between OX-*FZP* (*fzp-11*) and *fzp-11* at *P* < 0.01 (Student’s *t*-test).

**Table 2:** Number of floral organs in wild-type and OX-*FZP* plants.

Plants	Whorl 1			Whorl 2		Whorl 3		Whorl 4		Number of flowers examined
	Elongated empty glume^a^	Lemma	Palea	Lodicule	Status	Stamen^a^	Status	Carpel	Status	
ZH11	0	1	1	2	normal	6	normal	1	normal	20
Dongjin	0	1	1	2	normal	6	normal	1	normal	20
OX-*FZP*(ZH11)	1.1 ± 0.4*	1	1	2	enlarged	3.5 ± 1.9**	weak	1	enlarged	40
OX-*FZP*(*fzp-11*)	1.3 ± 0.3**	1	1	2	enlarged	1.9 ± 2.4**	weak	1	enlarged	40

Elongated empty glume^a^ and Stamen^a^, the data displayed by mean ± standard deviation. Significantly different from wild-type plant at *P < 0.05 and **P < 0.01 (Student’s *t*-test), respectively.
