# Supplementary material for: Regulatory role of FZP in the determination of panicle branching and spikelet formation in rice
Source: Sci Rep. 2016 Jan 8;6:19022. doi: 10.1038/srep19022 (PMC4705600; doi:10.1038/srep19022)
Supplement: Supplementary Information [file srep19022-s1.pdf]

Regulatory role of *FZP* in the determination of panicle branching and spikelet formation in rice

Xufeng Bai[1], Yong Huang[1] Donghai Mao[1], Mi Wen[1], Li Zhang[1] and Yongzhong Xing[1,2]*

Supplementary Figure legends

Figure S1

Structure of the *FZP* gene and the mutation sites of *fzp-11*

A, Structure of the *FZP* gene showing the mutation positions of different *fzp* mutants. The whole rectangle represents the entire open reading frame (ORF) of *FZP*, the AP2/ERF domain and the acidic domain are indicated by the black and gray rectangles.

B, The mutation sites of the *fzp-11* mutant identified by sequencing. Wt, Mt and H represent *FZP* wild type, mutant and heterozygote.

C, The positive transgenic plants of genetic complementation (T_0) identified by sequencing, the heterozygote (A/T) showed a positive phenotypes with normal panicles rescuing the mutant of *fzp-11*.

Figure S2

Phenotypes of wild type (Dongjin，DJ) and OX-*FZP* (*fzp-11*) plants

A, The leaf color in plants DJ (L) and OX-*FZP*-(*fzp-11*) (R). B, the transverse section of the stem of plants DJ (L) and OX-*FZP*-(*fzp-11*) (R). C-D, the stem transverse section under microscope, DJ plant (C) and OX-*FZP*-(*fzp-11*) plant (D). E, The leaf angle of plants DJ (L) and OX-*FZP*-(*fzp-11*) (R), the pulvinus are marked by red arrows. F, The cross section of the pulvinus of DJ (up) and OX-*FZP*-(*fzp-11*) (down). G, The root system of DJ (L) and OX-*FZP*-(*fzp-11*) (R). H and I, Inflorescence observations of wild-type (DJ) and OX-*FZP*-(*fzp-11*) by scanning electron microscopy, the apical meristem of inflorescence in OX-*FZP*-(*fzp-11*) is closed-up in the inset (I).

Scale bars, 3 cm (A), 0.5 cm (B), 300 μm (C and D), 1 cm (E), 3 mm (F), 2 cm (G).

Figure S3

Expression patterns of *RFL/APO2* and *LAX1*

A, The relative expression levels of *LAX1* both in YP1 and YP2 of plants from *fzp-11*, wild-type (DJ) and OX-*FZP*-(*fzp-11*). *Ubiquitin* served as the control. The bars indicate standard deviations.

B, RNA *in situ* hybridization analysis of *RFL/APO2* and *LAX1*. The YP1 of Mt (*fzp-11*), Wt (DJ) and OX (OX-*FZP*-(*fzp-11*)) showing branch meristems (BM) were used for

expression analysis. Blue arrows indicate the expressional areas of *RFL*/*APO2* similar to those of *FZP*, sense probe control is also showed. Scale bar = 100 μm.

C, RNA *in situ* hybridization analysis of *FZP*. Different young panicles of Wt (DJ) were used for expression analysis. Red arrows indicate expressional areas of *FZP*, sense probe control is also showed. Scale bar = 100 μm.

Figure S4

The relative expression analysis of panicle branching genes and floral identity genes

A, qRT-PCR analysis of *RFL*/*APO2* and *FZP* in the YP1 of OX-*FZP*-(ZH11) and CK (ZH11) plants.

B, qRT-PCR analysis of 13 investigated floral identity genes in the young panicles of YP1 and YP2 and FHP, respectively; Wt, wild type (DJ); Mt, mutant *fzp-11*.

The bars indicate standard deviations. The student *t*-test used for statistical analysis, *, ** Significant at the level of $P < 0.05$ and 0.01, respectively.

Figure S5

Expression patterns of the floral identity genes

RNA *in situ* hybridization analysis of *FZP*, *RFL*/*APO2*, *OsMADS4*, *OsMADS16*, *OsMADS58*, *OsMADS6*, *OsMADS17* and *OsMADS8*, the floral meristem (FM) form young panicles of Wt (DJ) plants were used for expression pattern analysis, red arrows indicate expressional areas for each gene; Le, lemma; Pa, palea.

Table S1 The primers were used for real-time PCR, RNA *in Situ* hybridization, sequencing and construction of vectors

Primer name	Sequence	
	Forward (5’-3’)	Reverse (5’-3’)
Ubiquitin-qRT	aaccagctgaggcccaaga	acgattgatttaaccagtccatga
LAX1-qRT	atgctggagcaggccatcca	gttcagctcaagggccaga
LAX1-in situ	ggcagcaagatggacacg	aagacacagcaaggcaaagg
FZP-qRT	cagggctccgactcctactct	caatgcccggtgcaactc
FZP-in situ	atgaacactcgaggcagc	ctcgctcatcggcgacga
SNB-qRT	gtccgggcttctttgtgaac	gggagccctgcatttgc
APO1-qRT	gacgtggcggagaacgta	ctgagacggctcttctcgac
OsMADS1-qRT	atctgagcaatgaagcagca	gcctgaagcctgaactgaac
OsMADS1-in situ	gaagagcaaggagcaacagc	gcctgaagcctgaactgaac
OsMADS2-qRT	gaagacgagaacaagctgct	ttcaatccatgggtggatca
OsMADS2-in situ	gggatgagaagcacaagagc	attgttctcctgcaggttgg
OsMADS3-qRT	gaaatctgaaggcgtcgatacc	cgaacacatagaattactgcatcgat
OsMADS3-in situ	agtcaatgcccagcactacc	gctgctgcatgatgttcact
OsMADS4-qRT	caatctgcgggacaagatg	gagctccagctcccttatgc
OsMADS4-in situ	gcagagatcgatcgtgtcaa	acttctcctgctggaggttg
OsMADS6-qRT	actacagagccatgcagcaa	cttcagcaggcacaaattga
OsMADS6-in situ	ggaggatcttggaccactca	gggcagtgctcctctgaata
OsMADS7-qRT	gagcagggctgcaacttaat	cgcttaaagttgatcgcaga
OsMADS7-in situ	agctcgagagcctagagaagc	atgggggcatgtaggtgtt
OsMADS8-qRT	caccttgcagatcgggttta	atctgtgtcgtcacatccgt
OsMADS8-in situ	ggcaacggattcttccattc	ctgtcacgtacggcctttta
OsMADS16-qRT	ggcaggtgacctactcgaag	gcatcgacattttgctcaag
OsMADS16-in situ	cggcaagtaccacgagttct	gttgtccacgaacccgaac
OsMADS17-qRT	tgctcaaccaccaccagata	ggccatcccatcacaaagt
OsMADS17-in situ	tcttgctggtggtgaacatc	tggttgagcattcagcactc
OsMADS58-qRT	gtcgccctcgtcgtcttct	tagtttccttcacgctgttgttg
OsMADS58-in situ	gatcaatgcccagcactacc	aggctgatgcatgatgttga
RFL-qRT	aggtgcaatccatggctaag	cgcgtagcagtgcacgtagt
RFL-in situ	aagaacgggctggactacct	agagctggcggagtctggt
S3	gatcaaccgggaccatcac	ggcacacaaatccaaacctc
COZP	aaaactgcaggatttgatcaacccctgcac	cgcggatccgcctagctcactctcactctca

Table S2 List of differentially expressed genes controlling panicle branching and floral identity in the mutant of *fzp-11*

Gene	LOC-ID	Folds	Functions	References
RFL	LOC_Os04g51000	9 +	Involvement in panicle branching	Kyozuka et al. 1998; Rao et al. 2008
LAX1	LOC_Os01g61480	6 +	Initiation/maintenance of axillary meristems	Komatsu et al. 2001; Komatsu et al. 2003
OsMADS2	LOC_Os01g66030	10 -	Stamen emergence and lodicule identity	Yadav et al. 2007; Yao et al. 2008
OsMADS4	LOC_Os05g34940	9 -	Lodicule and stamen identities	Yao et al. 2008
OsMADS16	LOC_Os06g49840	7 -	Carpels, lodicules and stamens identities	Nagasawa et al. 2003; Xiao et al. 2003
OsMADS58	LOC_Os05g11414	59-	Carpels, lodicules and stamens identities	Yamaguchi et al. 2006
OsMADS17	LOC_Os04g49150	75 -	Determining floral organ and meristem identities	Ohmori et al. 2009
OsMADS6	LOC_Os02g45770	68 -	Determining floral organ and meristem identities	Ohmori et al. 2009; Li et al. 2011
OsMADS7	LOC_Os08g41950	120 -	Loss of floral determinacy	Cui et al. 2010

upregulation (+) and downregulation (-), $P<0.05$.

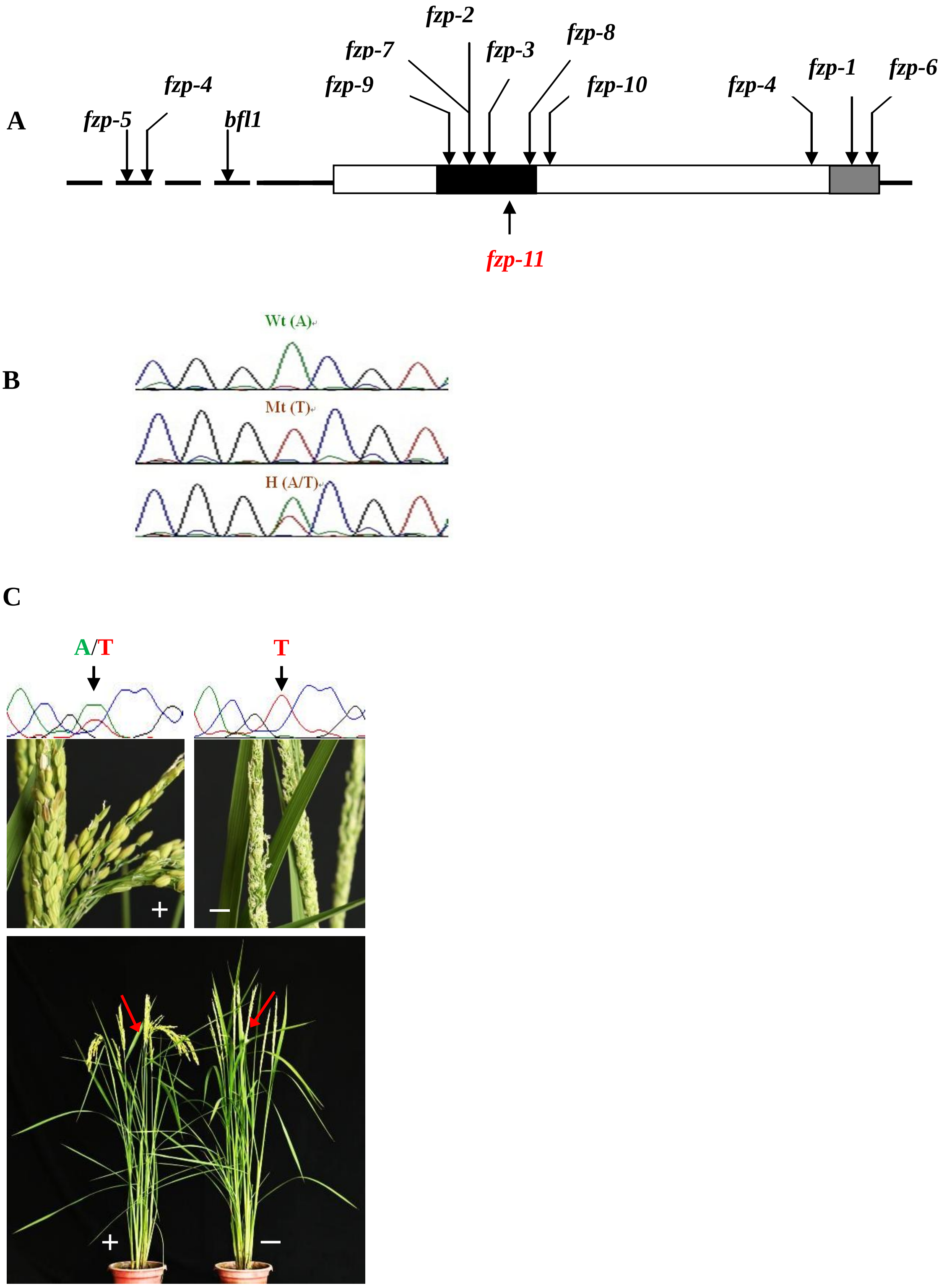
A
fzp-5
fzp-4
bfl1
fzp-9
fzp-7
fzp-2
fzp-3
fzp-8
fzp-10
fzp-4
fzp-1
fzp-6
fzp-11
B
Wt (A)
Mt (T)
H (A/T)
C
A/T
T
+
−
+
−

Figure S1

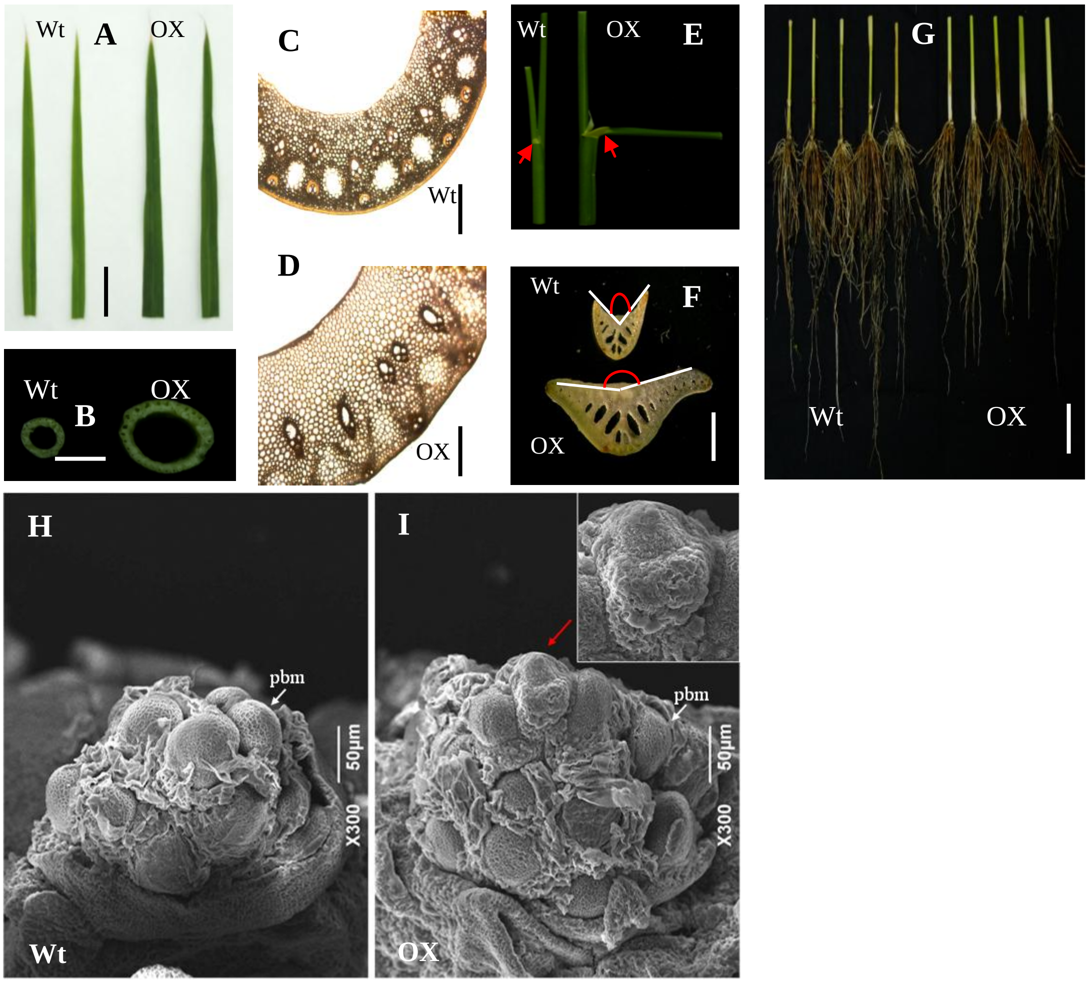

Figure S2

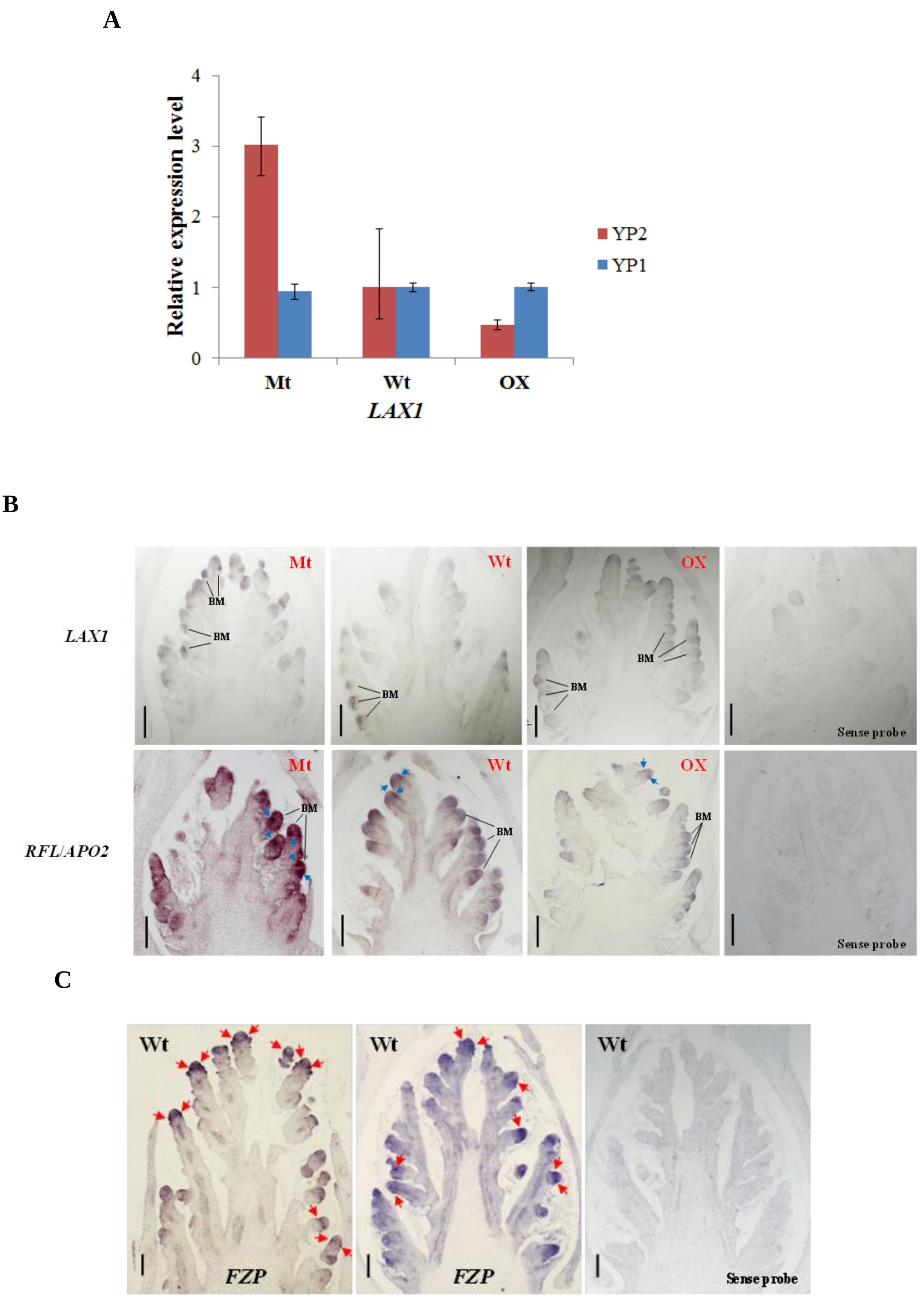
A
Relative expression level
4
3
2
1
0
Mt
Wt
OX
LAX1
YP2
YP1
B
LAX1
RFL/APO2
Mt
Wt
OX
BM
Sense probe
C
Wt
FZP
Sense probe

Figure S3

A

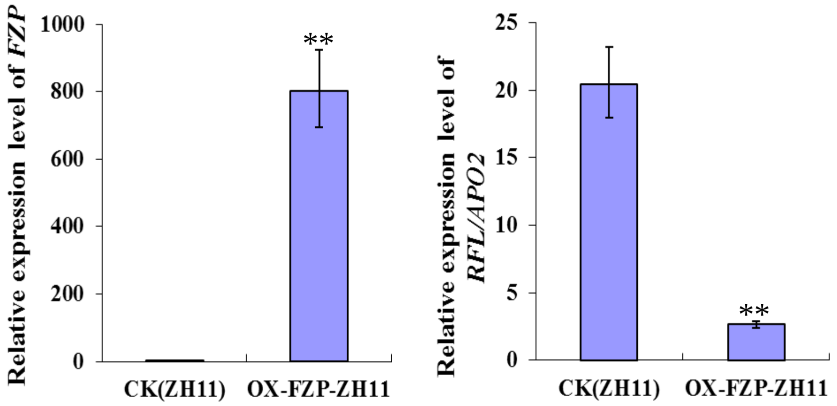
Relative expression level of FZP
1000
800
600
400
200
0
**
CK(ZH11)
OX-FZP-ZH11
Relative expression level of RFL/APO2
25
20
15
10
5
0
**
CK(ZH11)
OX-FZP-ZH11

B

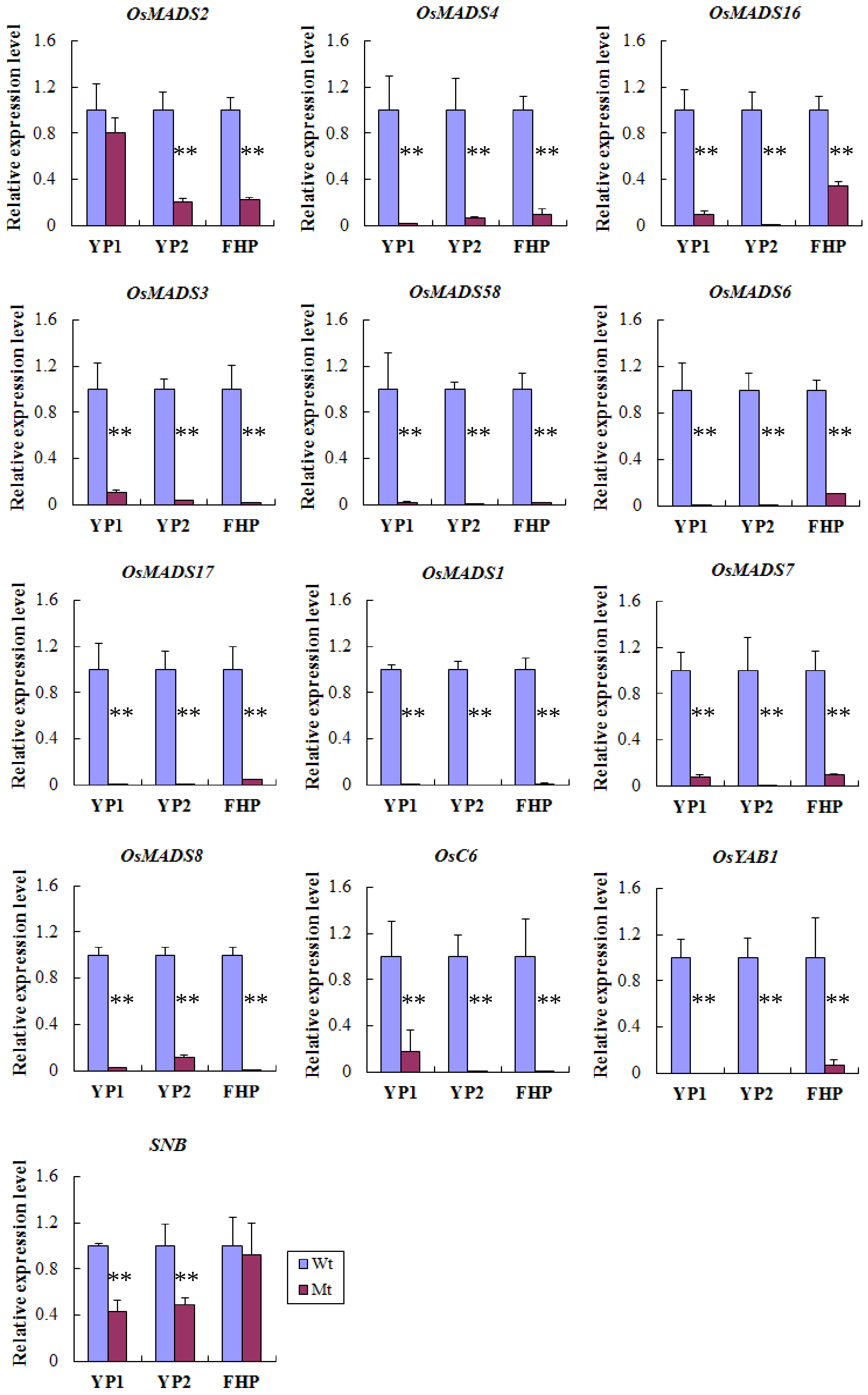
OsMADS2
OsMADS4
OsMADS16
OsMADS3
OsMADS58
OsMADS6
OsMADS17
OsMADS1
OsMADS7
OsMADS8
OsC6
OsYAB1
SNB
Relative expression level
0
0.4
0.8
1.2
1.6
YP1
YP2
FHP
**
Wt
Mt

Figure S4

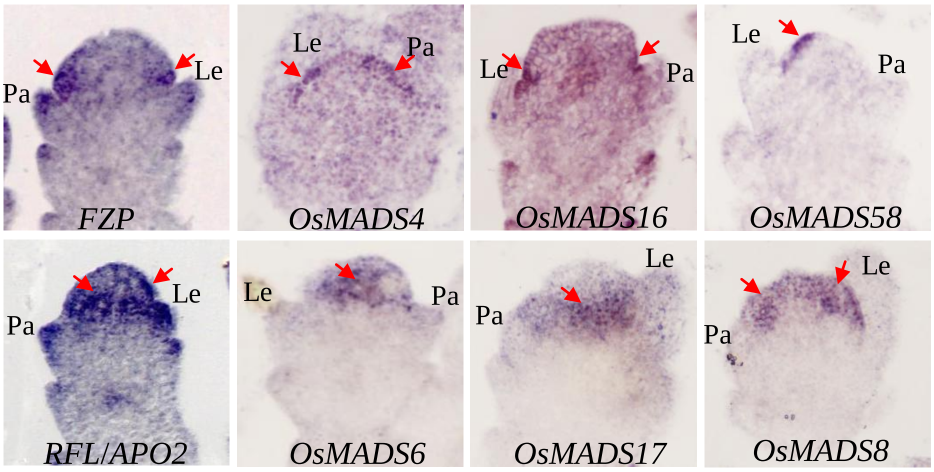
Pa
Le
FZP
Le
Pa
OsMADS4
Le
Pa
OsMADS16
Le
Pa
OsMADS58
Pa
Le
RFL/APO2
Le
Pa
OsMADS6
Le
Pa
OsMADS17
Le
Pa
OsMADS8

Figure S5
